# Genomic Analyses of >3,100 Nasopharyngeal Pneumococci Revealed Significant Differences Between Pneumococci Recovered in Four Different Geographical Regions

**DOI:** 10.3389/fmicb.2019.00317

**Published:** 2019-02-25

**Authors:** Andries J. van Tonder, James E. Bray, Keith A. Jolley, Melissa Jansen van Rensburg, Sigríður J. Quirk, Gunnsteinn Haraldsson, Martin C. J. Maiden, Stephen D. Bentley, Ásgeir Haraldsson, Helga Erlendsdóttir, Karl G. Kristinsson, Angela B. Brueggemann

**Affiliations:** ^1^Nuffield Department of Medicine, University of Oxford, Oxford, United Kingdom; ^2^Parasites and Microbes, Wellcome Sanger Institute, Hinxton, United Kingdom; ^3^Department of Zoology, University of Oxford, Oxford, United Kingdom; ^4^Department of Medicine, Imperial College London, London, United Kingdom; ^5^Clinical Microbiology, University of Iceland and Landspitali University Hospital, Reykjavik, Iceland; ^6^Institute of Infection and Global Health, University of Liverpool, Liverpool, United Kingdom; ^7^Department of Pathology, University of Cambridge, Cambridge, United Kingdom; ^8^Children’s Hospital Iceland, Landspitali University Hospital, Reykjavik, Iceland

**Keywords:** next generation sequencing, bacterial population structure, core genome, accessory genome, pan-genome, pneumococcus

## Abstract

Understanding the structure of a bacterial population is essential in order to understand bacterial evolution. Estimating the core genome (those genes common to all, or nearly all, strains of a species) is a key component of such analyses. The size and composition of the core genome varies by dataset, but we hypothesized that the variation between different collections of the same bacterial species would be minimal. To investigate this, we analyzed the genome sequences of 3,118 pneumococci recovered from healthy individuals in Reykjavik (Iceland), Southampton (United Kingdom), Boston (United States), and Maela (Thailand). The analyses revealed a “supercore” genome (genes shared by all 3,118 pneumococci) of 558 genes, although an additional 354 core genes were shared by pneumococci from Reykjavik, Southampton, and Boston. Overall, the size and composition of the core and pan-genomes among pneumococci recovered in Reykjavik, Southampton, and Boston were similar. Maela pneumococci were distinctly different in that they had a smaller core genome and larger pan-genome. The pan-genome of Maela pneumococci contained several >25 Kb sequence regions (flanked by pneumococcal genes) that were homologous to genomic regions found in other bacterial species. Overall, our work revealed that some subsets of the global pneumococcal population are highly heterogeneous, and our hypothesis was rejected. This is an important finding in terms of understanding genetic variation among pneumococci and is also an essential point of consideration before generalizing the findings from a single dataset to the wider pneumococcal population.

## Introduction

Collectively, the complete set of genes possessed by members of a bacterial species is defined as the pan-genome (Tettelin et al., 2005). Understanding bacterial population structure requires knowledge of which genes in the pan-genome are found in all, or nearly all, strains of that species (i.e., core genes), and which are only found in some strains (i.e., accessory genes). In any study, investigators characterize a subset of the global population and if one wishes to generalize the findings then it must be determined whether or not the single dataset is likely to be representative. Similarities and differences among datasets are equally interesting, for example: they provide the context for understanding bacterial population biology; they reveal the genetic lineages and which of those may be newly emerging, geographically restricted or internationally disseminated; they reveal local variation, adaptation and evolution among bacteria; and they provide clues about what determines the epidemiological success of individual genetic lineages.

We developed a Bayesian decision model for estimating the bacterial core genes for datasets comprised of incomplete (draft) genome sequences generated via next-generation sequencing methodologies (van Tonder et al., 2014). In that study we included the estimation of core genes for two different pneumococcal datasets, a diverse global historical dataset and a dataset of carriage genomes from Boston, MA, United States (Croucher et al., 2013). More recently, two additional genome datasets of carriage pneumococci recovered from healthy children in Southampton, United Kingdom, and from young children and their mothers living in the Maela refugee camp on the Thailand-Myanmar border were published (Chewapreecha et al., 2014; Gladstone et al., 2015). The genomes of pneumococci recovered from healthy young children recruited to our vaccine impact study in Iceland were also available, many of which have already been published (van Tonder et al., 2015, 2016; Quirk et al., 2018). Thus, four well-sampled datasets of pneumococci recovered from healthy individuals in four different geographical locations were available for comparison.

The aim of this study was to test the hypothesis that the estimated set of core genes within any one pneumococcal dataset accurately represents the estimated set of core genes among pneumococci recovered in other geographical locations. To achieve this, we analyzed four datasets of carriage pneumococci and: (i) estimated and compared the four sets of core genes; (ii) identified and characterized the shared “supercore” genome; and (iii) given the observed variation in the Maela dataset, assessed the genes that comprise the pan-genome of each dataset.

## Materials and Methods

### Pneumococcal Carriage Datasets Selected for Analyses

Icelandic pneumococci (*n* = 987) were recovered from the nasopharynx of healthy children 1–6 years old attending day care centers located in the greater capital area of Reykjavik, Kopavogur and Hafnarfjordur from 2009 to 2014 (van Tonder et al., 2015, 2016; Quirk et al., 2018). Pneumococci from Boston, MA, United States (*n* = 616) were recovered from the nasopharynx of healthy children <7 years old who were attending primary care facilities in and around Boston from 2001 to 2007 (Croucher et al., 2013). Pneumococci from the United Kingdom (*n* = 518) were isolated from the nasopharynx of children ≤4 years old attending the Southampton General Hospital outpatient department from 2006 to 2011 (Gladstone et al., 2015). Nasopharyngeal pneumococci (*n* = 3,085) from Maela, a refugee camp close to the border of Thailand and Myanmar, were collected from a cohort of 528 infants and 242 of their mothers from 2007 to 2010 as part of a longitudinal carriage study (Chewapreecha et al., 2014). The nasopharyngeal sampling was approved by ethics committees for each of the original studies (Chewapreecha et al., 2014; Gladstone et al., 2015; van Tonder et al., 2015, 2016; Quirk et al., 2018). All of the pneumococcal genomes were sequenced on the Illumina platform at the Sanger Institute.

Children in Reykjavik, Southampton and Boston were vaccinated with the 7-, 10-, or 13-valent pneumococcal conjugate vaccine (PCV) at some point before and/or after the time pneumococci were collected in each of the original studies. PCV10 was introduced into Iceland in 2011, PCV7 was used in the United Kingdom from 2006 to 2009 and PCV13 thereafter, and PCV7 was introduced in the United States in 2000 and was replaced by PCV13 in 2010 (Croucher et al., 2013; Gladstone et al., 2015; Moore et al., 2015; Quirk et al., 2018). No PCV was used in Thailand at the time pneumococci were collected in Maela.

Metadata for the Southampton, Boston, and Maela genome datasets were manually extracted from the original publications. Complete lists of the pneumococcal genomes included in this study, with accession numbers and available metadata are listed in Supplementary Table S1.

### Genome Assembly and Sequence Quality Assessment

The raw genome sequence data for all four datasets were downloaded from the European Nucleotide Archive, assembled using Velvet and uploaded to the ribosomal multilocus sequence typing (rMLST) database (Jolley et al., 2012). Genome sequence assemblies were assessed for total genome length and number of contigs, and rMLST loci were tagged to assign the bacterial species (Jolley et al., 2012). Only genome assemblies with a total length of 1.9–2.3 Mb and <500 contigs were included in this study. Among the Maela genome assemblies, 80 of the 3,085 genomes from the original dataset failed initial quality control due to them having non-pneumococcal rMLST profiles and these were discarded. 1,000 genomes from the original Maela dataset were randomly selected (using R; The R Foundation^1^) for inclusion in this study, to avoid bias due to the large size of the Maela dataset and to select a dataset similar in size to that of Reykjavik. Two genomes from the Southampton dataset were also removed from further analyses because those assemblies were too large to be consistent with the expected size of a pneumococcal genome. Finally, we compared the distribution of contigs assembled for all genome sequences and noted that there were differences between datasets, although the distributions of the contigs overlapped (Supplementary Figure S1): Reykjavik (range 25–356, median = 112 contigs); Southampton (range 52–248, median = 96 contigs), and Boston (range 50–246, median = 93 contigs); and Maela (range 83–475, median = 207 contigs). All assembled genomes are available for download from the *Streptococcus pneumoniae* PubMLST database^2^.

### Sampling Strategy for the Maela Dataset

After the initial data analyses revealed the Maela dataset to be more diverse than the other datasets, we assessed whether the random sampling strategy had biased the Maela dataset toward greater diversity. To do this we sampled another 1,000 genomes from the approximately 2,000 remaining genomes, however, the overall number of STs and serotypes for this sample were nearly identical to the original sample dataset (data not shown), suggesting that the observed epidemiological diversity in the study subset of 1,000 genomes was similar to that of the other Maela genomes not included here.

### Pneumococcal and Non-pneumococcal Species Comparison

1,000 genomes from 65 non-pneumococcal *Streptococcus* spp. were selected for comparative analyses to ensure that only pneumococci were included in this study (Supplementary Table S2). BIGSdb was used to extract the rMLST gene sequences from the 1,000 *Streptococcus* spp. genomes and the 3,118 pneumococcal genomes analyzed in this study. These rMLST sequences were aligned, concatenated and used to construct a phylogenetic tree (Katoh et al., 2002; Jolley and Maiden, 2010; Price et al., 2010). ClonalFrameML was used to reconstruct the tree and annotation was performed with iTOL (Letunic and Bork, 2011; Didelot and Wilson, 2015).

### Core Genome Analyses

Prokka (v 1.10) was used to predict and annotate the coding sequences, hereafter referred to as “genes” for simplicity, in each genome (Seemann, 2014). Gene annotation was based upon a bespoke pneumococcal sequence database compiled for this study, which used the gene annotation data from all available pneumococcal genomes in GenBank (National Center for Biotechnology Information Support Center^3^). The resulting annotation files in GFF format were input into Roary and clustered using sequence similarity thresholds of ≥70 and 90% (the ≥70% threshold to account for large nucleotide differences between the same gene in a population, e.g., nucleotide similarity of *pbp2x* may differ by ≥25% between penicillin-susceptible and -resistant pneumococci) (Page et al., 2015). Core genomes were calculated for each dataset using our Bayesian method (van Tonder et al., 2014). Putative paralogs were removed and the resulting core genes were extracted and aligned using MAFFT (Katoh et al., 2002).

Four dataset-specific sets of core gene sequences were created by extracting one amino acid sequence for every core gene in each dataset. The four sets of core genes were then compared and clustered in cd-hit using a similarity threshold of ≥90% and the “supercore” genome (the core genes that were present in every dataset) was determined (Li and Godzik, 2006). A Clusters of Orthologous Groups (COGs) functional category was assigned to each gene using eggNOG (Huerta-Cepas et al., 2016). Sequence alignments for the supercore genes were concatenated to create a supercore genome alignment that was used to create a phylogenetic tree using FastTreeMP (Price et al., 2010). The tree was reconstructed to account for recombination using ClonalFrameML (Didelot and Wilson, 2015). hierBAPS sequence clusters (SCs) were delineated and depicted on the final phylogenetic tree using iTOL (Letunic and Bork, 2011; Cheng et al., 2013).

### Pneumococcal Essential Genes

In a previously published study, Tn-seq was used to identify 397 pneumococcal genes that were likely to be essential for survival (van Opijnen et al., 2009). The relevant amino acid sequences for these genes were extracted from the TIGR4 genome and cd-hit was used to compare the amino acid sequences of the TIGR4 essential genes and the supercore genes identified in the current study at a sequence similarity threshold of ≥70%.

### Pan-Genome Analyses

The nucleotide gene sequences for each of the dataset-specific pan-genomes were clustered in cd-hit using a similarity threshold of ≥70 and 90% nucleotide sequence similarity and a sequence alignment threshold of ≥90%. The 4,606 gene clusters unique to the Maela pan-genome were manually inspected using the gene identifier numbers assigned by Prokka and the gene frequency information provided by the Roary output. The nucleotide sequence for each unique region was extracted using Artemis and both GenBank and the set of 1,000 non-pneumococcal genomes were queried to find homologous regions of sequence (Carver et al., 2008; Jolley et al., 2012). Putative transposons were annotated using the *CONJscan* module (Ambroset et al., 2015). Homologous regions were compared using diagrams created with EasyFig (Sullivan et al., 2011).

### Sequence Types (STs), Clonal Complexes (CCs), and Serotypes

Multilocus sequence type (MLST) data were auto-extracted from each genome using BIGSdb and STs were clustered into CCs using Phyloviz (Jolley and Maiden, 2010; Francisco et al., 2012). Our sequence-based serotyping programme seqSerotyper.R was used to assign serotypes based upon the nucleotide sequences of the capsular gene loci (van Tonder, 2016; van Tonder et al., 2016).

## Results

### Estimated Core Genome Comparisons

The study dataset was comprised of 3,118 genomes and each individual dataset represented a wide range of serotypes and clonal complexes (Table 1 and Supplementary Table S1). The number of dataset-specific core genes calculated for pneumococci recovered in Reykjavik (*n* = 1,112), Southampton (*n* = 1,135), and Boston (*n* = 1,108) were nearly identical, whereas there were only 671 estimated core genes among Maela pneumococci (Table 2). For comparison, the number of core genes in a highly diverse global and historical dataset of 336 pneumococci recovered from both carriage and disease was estimated to be 851 genes using the same Bayesian model (van Tonder et al., 2014). Such a diverse dataset would be expected to have a smaller set of shared core genes than a less diverse dataset from a single geographical location. The percentage of genomes in each dataset that possessed each estimated core gene ranged from ≥99.8 to 99.9%, which was consistent with the values calculated for other bacterial species datasets (van Tonder et al., 2014). The number of putative paralogues in any dataset was small (3–9 genes) and these were removed from further analyses.

**Table 1:** Summary of the pneumococcal genome datasets analyzed in this study.

Location	Genomes (n)	Years of isolation	STs^a^ (n)	CCs^b^ (n)	Serotypes (n)	PCV status^c^	Source of data
Reykjavik	986	2009–2014	98	42	31	Pre- and post-PCV10	van Tonder et al., 2015, 2016; Quirk et al., 2018
Southampton	516	2006–2011	128	54	43	Post-PCV7/13	Gladstone et al., 2015
Boston	616	2001–2007	139	56	31	Post-PCV7/13	Croucher et al., 2013
Maela	1,000	2004–2010	215	85	63	PCV naive	Chewapreecha et al., 2014


*^*a*^ST, multilocus sequence type.*

*^b^CC, clonal complex. The number of Singleton STs (those without any links to another ST) are not included in the total number of CCs, but are as follows: Reykjavik, n = 5; Southampton, n = 4; Boston, n = 3; Maela, n = 36.*

*^*c*^PCV, pneumococcal conjugate vaccine and 7, 10, and 13 refers to vaccine valency.*

**Table 2:** Summary of the estimated core genome and pan-genome for each pneumococcal genome dataset.

Location	% of genomes that possess each core gene	Putative paralogs (n)	Genes within estimated core genome (n)	Genes within pan-genome (n)
Reykjavik	≥99.8	9	1,112	7,340
Southampton	≥99.8	3	1,135	6,821
Boston	≥99.8	6	1,108	6,885
Maela	≥99.9	5	671	12,184


**FIGURE 1:**
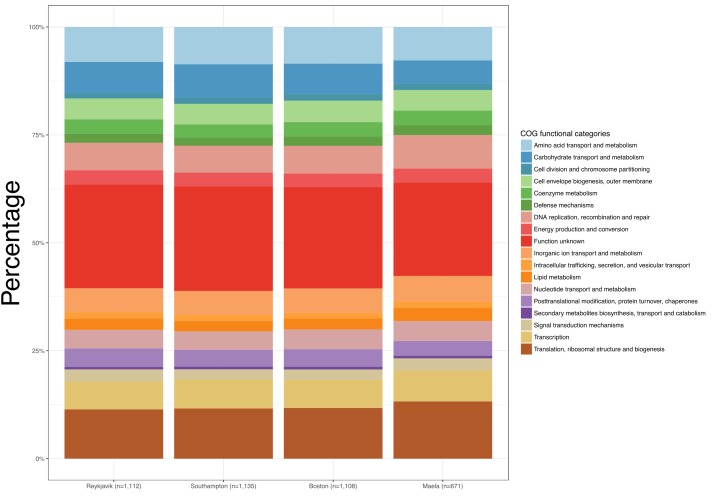
Bar graphs depicting the Clusters of Orthologous Groups (COGs) functional categories for each set of estimated core genes.

Despite the differences observed in the number of estimated core genes, the distribution of COGs functional categories among the core genes in each of the four datasets were similar (Figure 1 and Supplementary Table S3). In every case the largest proportion of estimated core genes were of unknown function (21.6–24.2%). As would be expected of core genes, the functional categories were those related to metabolism, transcription, translation, replication, recombination, cellular processes, and signaling.

### The Supercore Genome and Essential Genes

**FIGURE 2:**
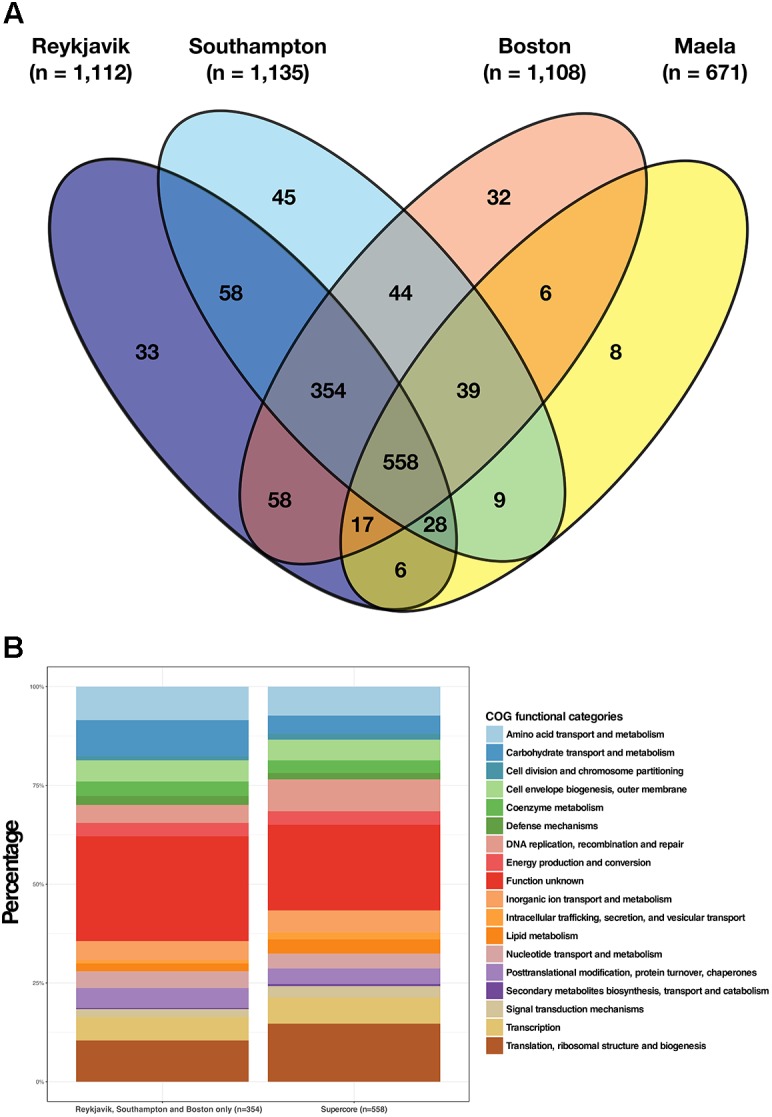
Illustration of the estimated core genes in each dataset and the shared supercore genome. **(A)** Venn diagram depicting the numbers of core genes shared by the four datasets and the 558 genes in the shared supercore genome; **(B)** COGs functional categories for the supercore genes as compared to the additional core genes shared by the Reykjavik, Southampton and Boston datasets only.

There were 558 estimated core genes shared by all four pneumococcal datasets and we defined these as the supercore genome (Figure 2A). A further 354 genes were shared by the Reykjavik, Southampton, and Boston pneumococci, thus there were 912 shared core genes in total between these three datasets (Supplementary Table S4). Examination of the 354 genes common to the Reykjavik, Southampton and Boston datasets revealed that the distribution of COGs functional categories broadly resembled that of the supercore genome (Figure 2B). Earlier work by van Opijnen and colleagues predicted that only 397 genes in an acapsular derivative of the TIGR4 pneumococcal genome were essential to fitness (van Opijnen et al., 2009) and 260 of these were amongst the supercore genes. The majority of these genes were involved in basic cell functions such as DNA replication, RNA transcription, translation, and central carbon metabolism.

### Supercore Genome Phylogeny

**FIGURE 3:**
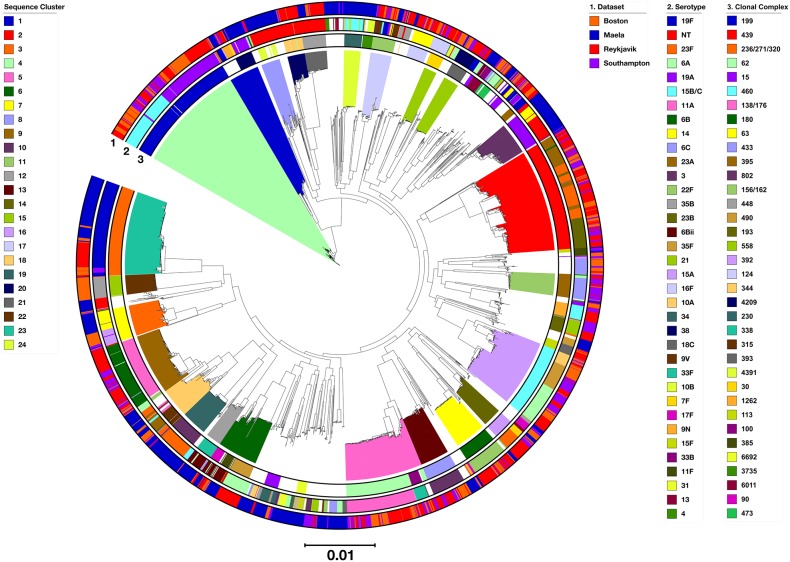
Phylogenetic tree representing the relationships among all 3,118 pneumococci, constructed based on the concatenated sequence alignment of 558 supercore genes. Clades were colored according to hierBAPS sequence cluster. Outer rings were annotated and colored as depicted in the legends. The serotypes and CCs represented by >10 and >20 genomes, respectively, were annotated here.

All 3,118 genomes were represented by a phylogenetic tree constructed using the 558 supercore gene sequences clustered with hierBAPS (Figure 3). The hierBAPS analysis revealed 24 monophyletic SCs that ranged in size from 37 to 263 genomes and were concordant with clonal complexes defined using MLST data (Figure 3 and Supplementary Table S5). Pneumococci representing eight clonal complexes were found in all four locations (CC^predominant serotype(s)^): CC138/176^6B^ (*n* = 145; SC9 and SC18); CC15^14,NT^ (*n* = 131; SC10 and unassigned); CC156/162^9V,19F^ (*n* = 58; SC9 and SC17); CC448^NT^ (*n* = 52; SC21); CC344^NT^ (*n* = 41; SC20); CC338^23F/A/B^ (*n* = 37; SC18); CC393^38^ (*n* = 37; SC15); and CC113^18C,31^ (*n* = 26; SC unassigned). All of these are widely-distributed genetic lineages (The PubMLST database^4^).

Pneumococci from other widely-distributed clonal complexes were identified in Reykjavik, Southampton and Boston, but not Maela: CC199^19A,15B/C^ (*n* = 260; SC4); CC439^23F/A/B^ (*n* = 244; SC2); CC460^6A^ (*n* = 159; SC16); CC62^11A^ (*n* = 158; SC5); CC180^3^ (*n* = 74; SC7); CC433^22F^ (*n* = 69; SC13); CC395^6C^ (*n* = 67; SC11); CC392^23F,17F^ (*n* = 45; SC14); CC124^14^ (*n* = 42; SC unassigned); CC30^16F^ (*n* = 31; SC15); CC100^33F^ (*n* = 24; SC5); CC473^6A/C^ (*n* = 21; SC unassigned); CC1379^6C^ (*n* = 17; SC unassigned); CC66^9N^ (*n* = 17; SC1); CC432^21^ (*n* = 14; SC unassigned); CC452^35B^ (*n* = 9; SC unassigned); and CC568^31^ (*n* = 6; SC unassigned).

Pneumococci representing CC236/271/320^19F/A^ (*n* = 200; SC23), CC315^6Bii^ (*n* = 37; SC12), CC90^6Bii^ (*n* = 20; SC6), and CC171^23F^ (*n* = 15; SC9 and SC18), three of which are multidrug-resistant clonal complexes (CC171^23F^ is the exception), were found in all locations apart from Southampton. CC63^14,15A^ (*n* = 73; SC9), CC230^19A^ (*n* = 38; SC1), CC81^19F,23F^ (*n* = 16; SC1), CC218^varied^ (*n* = 7; SC not assigned), and CC242^23F^ (*n* = 6; SC not assigned) were found in all locations apart from Reykjavik. These are also widely-distributed genetic lineages (The PubMLST database^4^).

The hierBAPS analysis also identified 854 genomes that were represented by polyphyletic sequence clusters (uncolored clusters, Figure 3). 42.5% of all Maela genomes were within polyphyletic sequence clusters, in contrast to <25% of genomes in the datasets from Reykjavik (16.7%), Southampton (23.5%), and Boston (23.2%).

### Pan-Genome Comparisons

**FIGURE 4:**
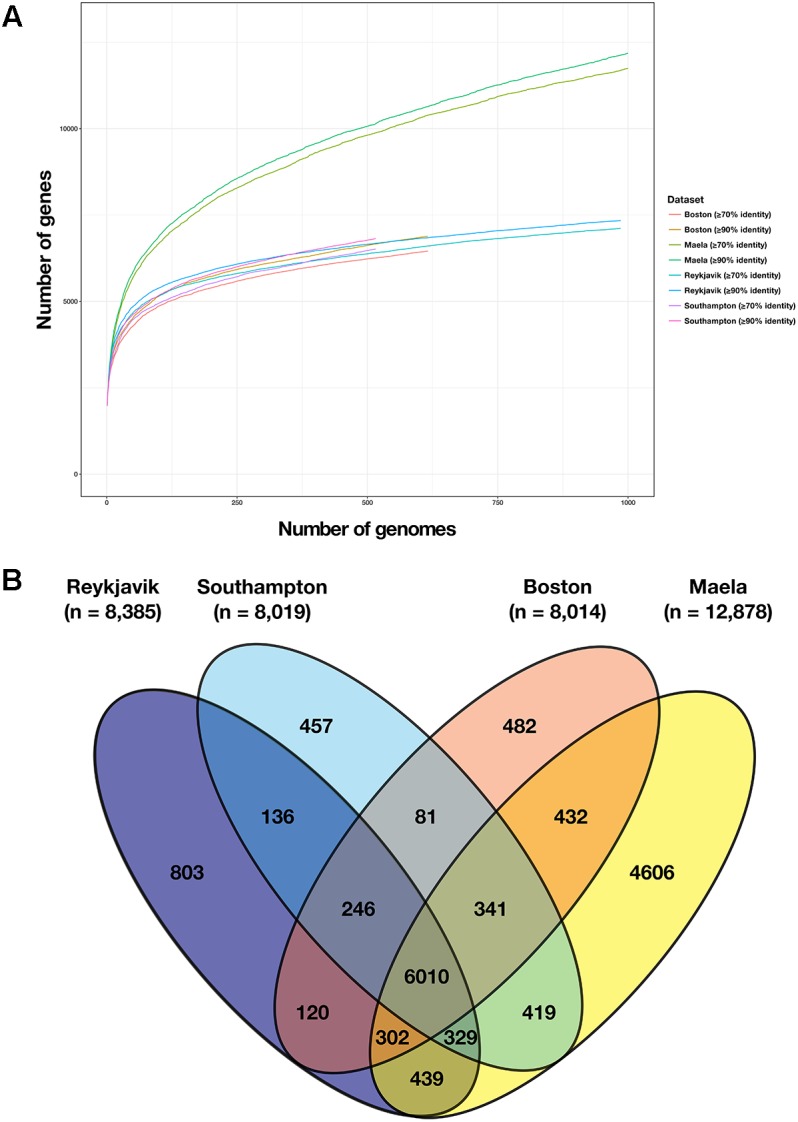
Comparison of the number of genes in the pan-genome of each dataset. **(A)** Results of Roary pan-genome analyses using two different thresholds, ≥70 and ≥90% nucleotide sequence similarity, for each of the four datasets; **(B)** Venn diagram depicting the number of genes present in the pan-genome of each dataset and of those, which were shared between datasets (using the ≥70% sequence similarity threshold).

The pan-genomes of the Reykjavik, Southampton, and Boston pneumococci were very similar in size (6,821–7,340 genes), in contrast to the 12,184 genes in the Maela pneumococcal pan-genome (Table 2). The dataset-specific pan-genomes were calculated twice for each dataset, using ≥70 and 90% nucleotide sequence similarity thresholds, however, there were minimal within-dataset differences in the total number of genes in the pan-genomes using either threshold (Figure 4A). The total number of genes in the pan-genomes of the Reykjavik, Southampton, and Boston datasets plateaued around 6,000–7,000 genes, whilst the Maela pan-genome continued to increase. All four pan-genomes were open, i.e., the number of genes increased as more genomes were added to the analysis, which was a previously reported observation in pneumococcal pan-genome analyses (Tettelin et al., 2005).

**FIGURE 5:**
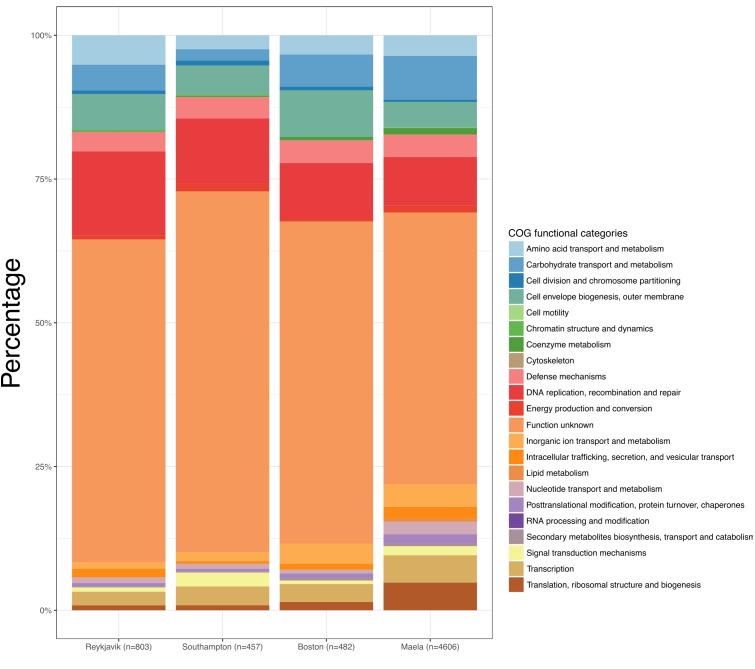
Bar graphs depicting the Clusters of Orthologous Groups (COGs) functional categories of the unique genes in each dataset.

Overall, among the four datasets there were 37,754 genes in the combined pan-genome and these formed 10,836 gene clusters at a threshold of ≥70% nucleotide sequence similarity. 6,010 of these gene clusters were identified among at least one pneumococcus from each of the four datasets (Figure 4B). The number of unique gene clusters in the Reykjavik (*n* = 803), Southampton (*n* = 457), and Boston (*n* = 482) datasets were broadly similar, as compared to 4,606 gene clusters unique to the Maela dataset. The function of approximately half of the unique gene clusters in any dataset was unknown (47.3–62.8%; Figure 5 and Supplementary Table S6).

### Potential Influence of Nontypable Pneumococci or Non-pneumococcal *Streptococcus* spp. Genomes

Nontypable pneumococci comprised 16.6% (*n* = 512) of the original Maela dataset, as compared to ≤6.6% of each of the three other datasets, and nontypable pneumococci are recognized as being a diverse group (Keller et al., 2016). To test whether the inclusion of a large proportion of nontypable pneumococci were strongly influencing the findings, the nontypable pneumococci were excluded from the full Maela dataset, a random sample of 1,000 genomes was selected from the remaining genomes and the core genome and pan-genome were recalculated. Exclusion of the nontypable genomes had a relatively minor effect on the size of the Maela pan-genome (decreased from 12,184 to 10,712 genes) and the estimated core genome (increased from 671 to 728 genes).

**FIGURE 6:**
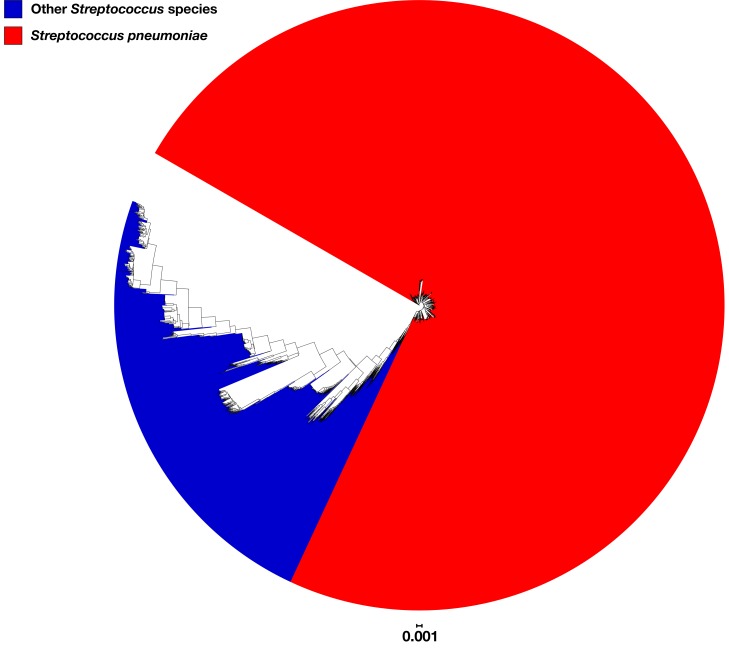
Phylogenetic tree constructed based upon the sequences of 53 ribosomal gene sequences extracted from the 3,118 pneumococcal study genomes plus 1,000 genomes of 65 different non-pneumococcal *Streptococcus* spp. genomes. Pneumococcal and non-pneumococcal clusters are colored red and blue, respectively.

Another possible explanation for the observed differences was that the Maela dataset contained genomes from non-pneumococcal *Streptococcus* spp. To investigate this possibility, a phylogenetic tree was constructed based upon the 53 rMLST loci sequences extracted from the 3,118 pneumococcal genomes plus 1,000 genomes of 65 different non-pneumococcal *Streptococcus* spp. (Supplementary Table S2; Jolley et al., 2012). All 3,118 pneumococcal genomes clustered together, separate from the non-pneumococcal *Streptococcus* spp. (Figure 6); therefore, the observed differences among the Maela dataset were unlikely to be explained by the inclusion of non-pneumococcal genomes.

### Large Unique Gene Regions in the Maela Pan-Genome

Although the Maela dataset was comprised of bona fide pneumococci, the large number of accessory genes unique to the Maela dataset suggested that genomic regions that were non-pneumococcal in origin might be influencing the results. Gene names and genomic positions for the 4,606 gene clusters unique to the Maela pan-genome were extracted and manually inspected to identify large (>25 Kb) genomic regions within contigs that contained genes of pneumococcal origin. Fourteen regions that ranged from 25.2 to 66.1 Kb were revealed (comprising 15% of all Maela unique genes), most of which were identified in multiple Maela genomes (Table 3). The nucleotide sequences of these 14 regions were extracted and used to query GenBank and the dataset of 1,000 non-pneumococcal *Streptococcus* spp. genomes to identify possible matches.

**Table 3:** Large genomic regions that were unique to the Maela dataset.

Representative genome	Length of region (bp)	No. of Maela genomes with region	GenBank best match (% identity)	*Streptococcus* spp. best match (% identity)	Fragment type
SMRU1398	66,142	7	***S. agalactiae* ILRI112 (98.0)**	*S. dysgalactiae* 66090 (96.4)	Tn*1549* and Tn*916* with *tet*(M)
SMRU1170	59,943 (1 gap)	2	***Filofactor alocis* ATCC35896 (99.0)**	*S. dysgalactiae* 65857 (89.8)	Tn*1549* and Tn*916* with *tet*(M)
SMRU1457	51,873	11	No significant match	***S. dysgalactiae* 65857 (97.4)**	Tn*1549* and Tn*916* with *tet*(M)
SMRU2268	41,961	2	***S. anginosus* C238 (93.0)**	*S. constellatus* 63991 (90.7)	Tn*1549* no Tn*916*
SMRU1351	39520	11	No significant match	No significant match	Prophage
SMRU2725	38,392	19	No significant match	No significant match	Unknown transposon fragment
SMRU392	34256	1	No significant match	No significant match	Prophage
SMRU158	32,902 (2 gaps)	1	*Streptococcus* sp. VT162 (94.0)	***S. oralis* 63998 (95.6)**	Unknown transposon fragment
SMRU1017	32,098	1	No significant match	No significant match	Partial prophage sequence
SMRU128	30,967	46	***S. pneumoniae* 70585 (99.0)**	*S. suis* 66662 (87.6)	Pentose and glucoronate interconversion region
SMRU148	30,628	1	No significant match	***S. oralis* ATCC42996 (94.9)**	TnGBS2
SMRU1266	28998	1	No significant match	No significant match	Partial prophage sequence
SMRU1770	26,625 (3 gaps)	3	*Streptococcus* sp. VT162 (85.0)	***S. pseudopneumoniae* 110329 (98.5)**	Unknown transposon fragment
SMRU602	25,230	1	***Staphylococcus aureus* 2395 USA500 (99.8)**	*S. dysgalactiae* 66058 (99.0)	Tn*916* with *tet*(M)


*The best overall significant matches are highlighted in bold.*

**FIGURE 7:**
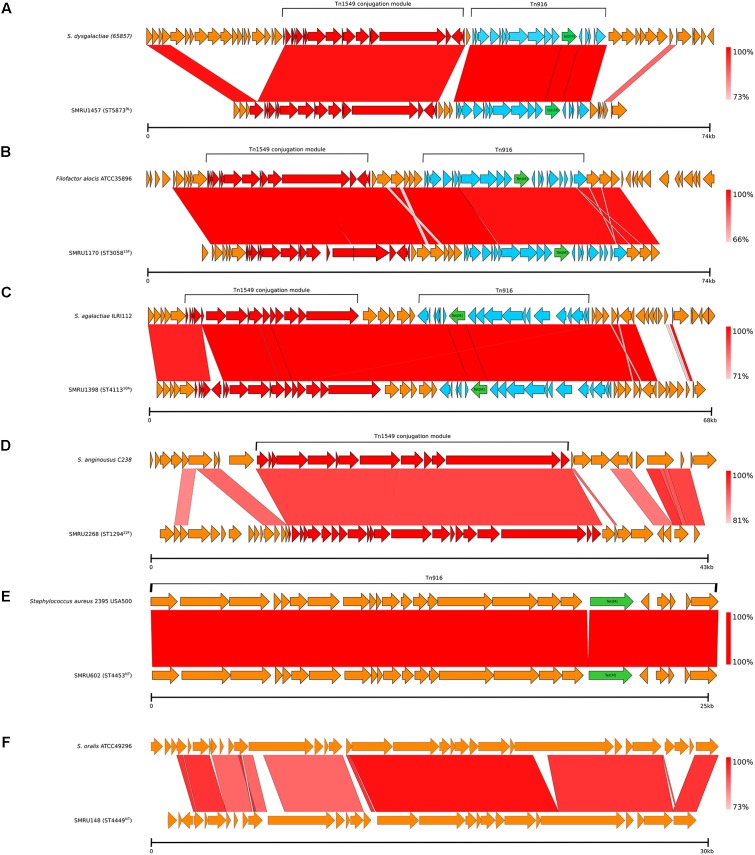
Mobile genetic elements identified among the Maela genomes. **(A–C)** Tn*1549*-like ICE with Tn*916*; **(D)** Tn*1549*-like ICE without Tn*916*; **(E)** Tn*916*; and **(F)** Tn*GBS2*.

Four different examples of Tn*1549*-like integrative and conjugative elements (ICEs), three of which included Tn*916* with *tet*(M), which mediates tetracycline resistance, were identified in 21 genomes (Table 3 and Figure 7). The nearest matches to these variable Tn*1549*-like regions were predominately non-pneumococcal *Streptococcus* spp., but a nearly identical match to the Tn*1549* region in one pneumococcal genome was from *Filofactor alocis* ATCC35896, a Gram-positive anaerobe implicated in periodontal disease (Aruni et al., 2015). Tn*916* was also found on its own in one Maela genome and it was identical to a Tn*916* in the *Staphylococcus aureus* 2395 USA500 genome. Tn*GBS2*, another type of ICE, was found in a single Maela genome. Tn*GBS2* uses a DDE transposase instead of a phage-like integrase for mobility and is found in oral *Streptococcus* spp. such as *S. mitis* and *S. oralis* (Guerillot et al., 2013).

No significant matches were identified in either GenBank or the *Streptococcus* spp. genomes for four different prophage sequences (28.9–39.5 Kb) and an unknown transposon fragment. The four prophage sequences were compared to our database of pneumococcal prophage sequences assembled from another collection of diverse pneumococcal genomes. Two of the Maela sequences were of two different, putatively full-length novel prophages that did not match to any previously reported prophages but were related to one cluster E prophage from our recent study (Brueggemann et al., 2017). These were submitted to GenBank (accession numbers MK044828 and MK044829). The two other putative prophage sequences were of incomplete prophages with no close matches in our database.

46 genomes from multiple genetic lineages possessed a 31.0 Kb region that contained a number of genes involved in carbohydrate metabolism, including PTS lactose and ascorbate transporters, and genes that constituted the pentose and glucuronate interconversion pathway, which is an alternative to glycolysis (Yew and Gerlt, 2002). A GenBank search revealed a nearly identical hit to *S. pneumoniae* 70585 (ST289^5^), a disease-causing pneumococcus from Bangladesh.

## Discussion

The relative ease with which bacterial genomes can be sequenced using next-generation sequencing technologies has resulted in a paradigm shift in our understanding of bacterial populations. MLST was developed 20 years ago and it quickly became a powerful tool for defining bacterial lineages. The explosion of MLST data fundamentally informed our understanding of bacterial population structure, recombination, evolution, epidemiology, pathogenesis, and the consequences of perturbing bacterial populations with vaccines.

Genomics now provides the data with which one can address hypotheses with a much higher resolution than ever before. Genomic analyses have not abrogated the relevance of MLST, but in fact genome sequence-based clustering (at least in the case of pneumococci) is highly concordant to clustering based on MLST data. This is helpful, since the MLST nomenclature is even more valuable with the overlay of genome-wide information, and as a result it is becoming clear that the diversity within some bacterial populations may be even more nuanced than previously appreciated.

Understanding the variation among different pneumococcal datasets is crucial, because genomic data are increasingly being analyzed in many different ways. The core genes common to all pneumococci are important to assess in the context of identifying potential vaccine candidates, population biology analyses, and more precisely defining genetic lineages beyond MLST loci. Importantly, the findings of one study must be shown to be reproducible and generalizable if they are to be extrapolated to the wider pneumococcal population. Prior to the availability of 1000s of genomes for analyses, core genes were defined as those genes present in all strains of a species, based upon a small set of fully-sequenced, closed genomes. The vast majority of genome sequences available today are “draft” genomes, i.e., a set of contigs, not a closed genome, and that requires a different approach to define the core genes, but one which must still be achieved in an unbiased manner (van Tonder et al., 2014). This study is about defining which genes are common among pneumococci and asking whether these common (core) genes are the largely the same (or not) among pneumococci from different geographical regions. In other words, here we are comparing large collections of pneumococci to identify which genes are common among pneumococci at the wider population level.

Our study clearly showed that the core and pan-genomes of geographically-distinct datasets of carried pneumococci from Reykjavik, Southampton, and Boston were similar in size and composition. In contrast, the dataset from Maela was unique in terms of its large pan-genome and small core genome, as well as the overall diversity of its CC and serotype distributions. Maela is a refugee camp of only ∼50,000 inhabitants and the movement of people in and out of the camp is restricted; therefore, our expectation was that there would be a similar bottleneck in the flow of pneumococci in and out of the camp, leading to a comparatively less diverse pneumococcal population. This was not observed – rather the diversity of pneumococci circulating in the Maela refugee camp was greater than that in any of the cities of Reykjavik, Southampton, and Boston. There were twice as many serotypes and more than double the number of CCs among the carriage pneumococci in Maela than there were in Reykjavik, which was the least diverse of the four datasets based on those criteria.

There were up to ten times as many unique gene clusters in the Maela pan-genome as there were in the other datasets and the majority of these unique clusters were found repeatedly in the Maela dataset. The large regions that were identified were predominately also found in other *Streptococcus* spp., but in two cases the best matches were to non-streptococcal bacteria. It seems likely that there are other genomic regions of interest to be found in the list of ∼4,600 genes unique to the Maela pan-genome, thus a more in-depth study of these regions and any other large regions identified in the pan-genomes of the other three datasets should be performed in future work.

Altogether, these findings suggest two important points: first, that the Maela genome data are not generalizable, and secondly, that the global pneumococcal population is likely to be more heterogeneous than currently appreciated, particular among those pneumococci from geographical regions that have never or rarely been sampled to date. The majority of epidemiological studies that have been conducted in developed countries have shown that the pneumococcal serotypes and STs that circulate in carriage and disease are broadly similar across different populations (Moore et al., 2015). In contrast, recent epidemiological studies in places like Bolivia, Kenya, Malaysia, and Nepal, which characterized pneumococci only by traditional MLST, demonstrated that whilst the most prevalent serotypes tend to be the same as those in high-income countries, the diversity of STs/CCs was greater (Inverarity et al., 2011; Brueggemann et al., 2013; Hanieh et al., 2014; Jefferies et al., 2014).

It is also important to be aware of differences that might exist between pneumococci recovered from carriage versus those recovered from disease and thus it is essential to ensure that comparative studies are using well-matched datasets of pneumococci (e.g., here we focussed only on carriage pneumococci) to avoid potential biases in the findings. Overall, our findings suggest that caution should be exercised and generalizability should be rigorously assessed before extrapolating broader biological conclusions about the pneumococcus based on a dataset from a narrow population sampling.

Importantly, safe and effective PCVs are now used in many countries, but they significantly disrupt the pneumococcal population structure and this can have unpredictable consequences (Brueggemann et al., 2007; Weinberger et al., 2011; Croucher et al., 2013). Therefore, characterizing the pre- and post-PCV pneumococcal population structure is essential in order to identify the changes that occur. Whilst traditional MLST is still highly useful in that regard and will remain the genotyping method of choice in many parts of the world for some time, genomics adds a higher discriminatory level of resolution to such analyses and should be employed wherever possible.

The availability of 1000s of bacterial genomes means that meta-analyses of large datasets can now be undertaken in order to more precisely delineate bacterial population structure and the composition of the bacterial population in terms of the pan-genome, core, and accessory genomes. More studies like this one will need to be carried out in order to better understand the heterogeneity of the global pneumococcal population in particular, and of bacterial populations more generally.

## Data Availability

The assembled genome sequences and corresponding metadata are available from the PubMLST website (https://pubmlst.org/spneumoniae/) and raw genome sequence data are via the NCBI Sequence Read Archive (see Supplementary Table S1 for accession numbers). The two prophage genomes may be found in GenBank (accession numbers MK044828 and MK044829).

## Author Contributions

AT and AB conceived and designed the study. SQ, GH, ÁH, HE, and KK collected and processed the Icelandic pneumococci. AT extracted DNA from the Icelandic pneumococci. SB sequenced the Icelandic pneumococcal genomes. JB and KJ assembled bacterial genomes in the rMLST databases. JB, KJ, MM, and AB managed and/or curated the rMLST and/or PubMLST databases. AT, MR, and AB performed the analyses. AT and AB wrote the manuscript. All authors read and approved the final manuscript.

## Conflict of Interest Statement

The authors declare that the research was conducted in the absence of any commercial or financial relationships that could be construed as a potential conflict of interest.

## Footnotes

1 https://www.R-project.org/

2 http://pubmlst.org/spneumoniae/

3 https://www.ncbi.nlm.nih.gov/genome/annotation_prok/

4 http://pubmlst.org/spneumoniae/

## Supplementary Material

FIGURE S1 Comparison of the four study datasets in terms of the number of assembled contigs in each genome assembly.

TABLE S1 Metadata and accession numbers associated with the 3,100 pneumococcal genomes analyzed in this study.

TABLE S2 Metadata and accession numbers associated with the 1,000 non-pneumococcal *Streptococcus* spp. analyzed in this study.

TABLE S3 Core genes identified in each of the four individual datasets.

TABLE S4 Supercore genes found in all pneumococcal study genomes, plus the additional core genes shared by the Reykjavik, Southampton, and Boston pneumococci.

TABLE S5 Associations between clonal complexes as defined by MLST and sequence clusters as defined by hierBAPS, stratified by geographical location.

TABLE S6 Unique gene clusters identified in each of the four individual datasets.
