# Supplementary figures and images for: Genomic Analyses of >3,100 Nasopharyngeal Pneumococci Revealed Significant Differences Between Pneumococci Recovered in Four Different Geographical Regions
Source: Front Microbiol. 2019 Feb 25;10:317. doi: 10.3389/fmicb.2019.00317 (PMC6398412; doi:10.3389/fmicb.2019.00317)

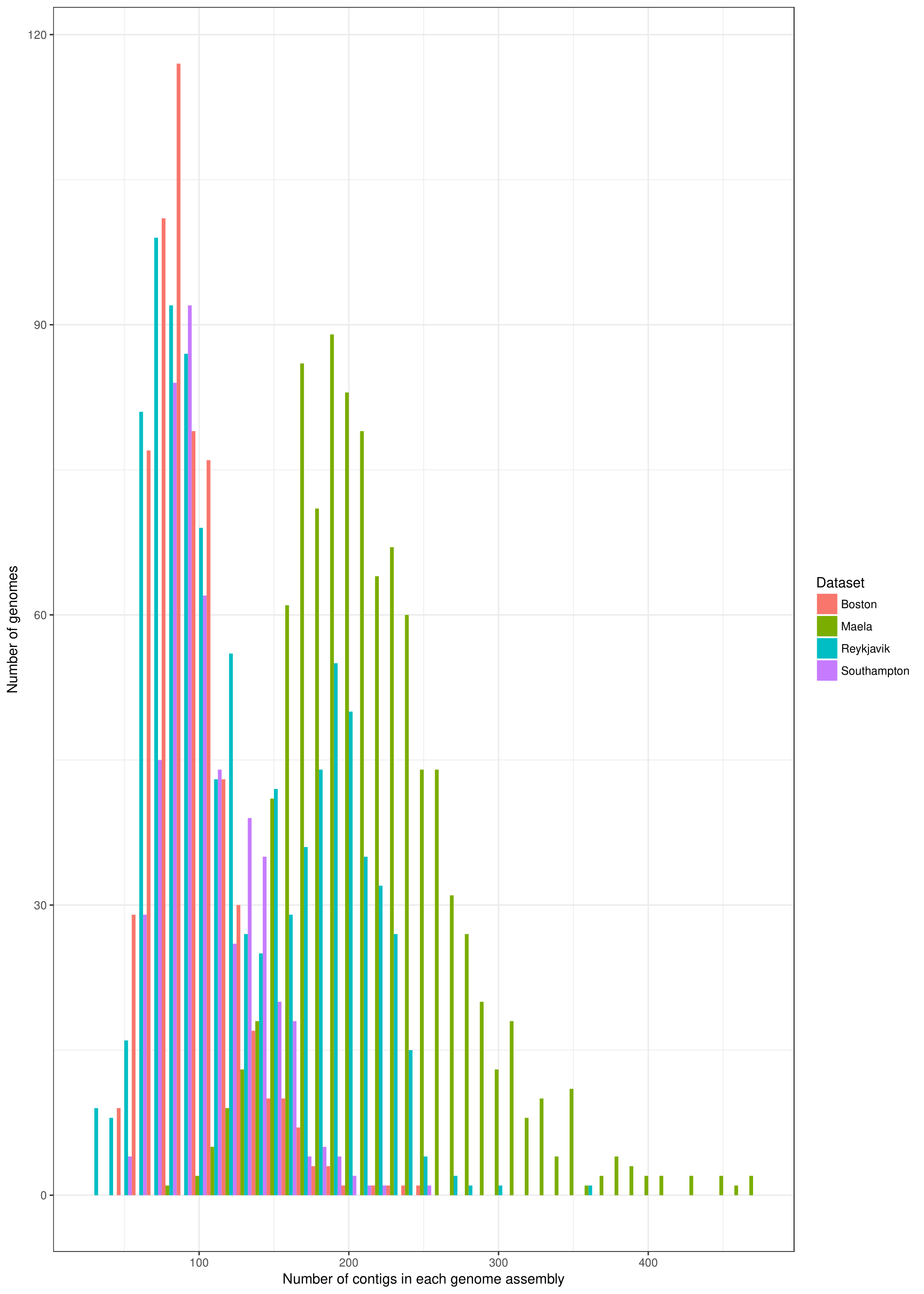

Supplement: FIGURE S1 — Comparison of the four study datasets in terms of the number of assembled contigs in each genome assembly. [file Image_1.TIFF]
